# Anesthetic and Transfusion Management of an Immunosuppressed Patient With Infective Endocarditis and Severe Platelet Allergy Undergoing Redo Aortic Root Surgery

**DOI:** 10.7759/cureus.83553

**Published:** 2025-05-06

**Authors:** Despoina Sarridou, Rafail Ioannidis, Foteini Iatridi, Giakoumis Mitos, Ekaterini Amaniti

**Affiliations:** 1 Department of Anesthesiology, American Hellenic Educational Progressive Association (AHEPA) University Hospital, Aristotle University of Thessaloniki, Thessaloniki, GRC; 2 Department of Anesthesiology and Pain Medicine, General Hospital of Drama, Democritus University of Thrace, Drama, GRC; 3 1st Department of Nephrology, Aristotle University of Thessaloniki, Thessaloniki, GRC; 4 Department of Anesthesiology and Intensive Care, Faculty of Medicine, School of Health Sciences, American Hellenic Educational Progressive Association (AHEPA) University Hospital, Aristotle University of Thessaloniki, Thessaloniki, GRC

**Keywords:** abcess, allergy, aortic root, lymphoma, platelets

## Abstract

Aortic root replacement for infected endocarditis is associated with excessive bleeding and very high mortality. Bleeding management is mostly achieved with allogenic blood product administration, concomitant pharmacologic agents, and application of hemostatic factors. An immunosuppressed male patient with large B-cell Lymphoma and thrombocytopenia with aortic root abscess underwent root replacement surgery with a homograft. After an allergic reaction to platelet transfusion, a hematologic consult was requested, and guided transfusion was performed perioperatively. In total, six pools of washed platelets and cryoprecipitate were administered. Fresh frozen plasma (FFP) was also used after a test dose. Patients with large B-cell lymphoma typically suffer from bone marrow suppression. An individualized approach is necessary to guide transfusion in this special population. The administration of FFP as a test dose before entering the cardiopulmonary bypass may be useful in managing hemodynamic compromise in case of a reaction. Multidisciplinary management led to a successful outcome.

## Introduction

Cardiac surgery involving aortic root replacement for infected endocarditis is associated with excessive bleeding, prolonged critical care stay, and high mortality. Bleeding management is achieved with the administration of allogenic blood products, pharmacologic agents, and hemostatic factors, such as tranexamic acid, fresh frozen plasma (FFP), and cryoprecipitate [1]. Our case involved a 54-year-old immunosuppressed male patient with large B-cell lymphoma and preoperative thrombocytopenia with aortic root abscess who underwent root replacement surgery with a homograft. Preoperatively, he developed a severe allergic reaction following platelet transfusion. These circumstances created huge limitations to the common transfusion strategies.

## Case presentation

**Figure 1:**
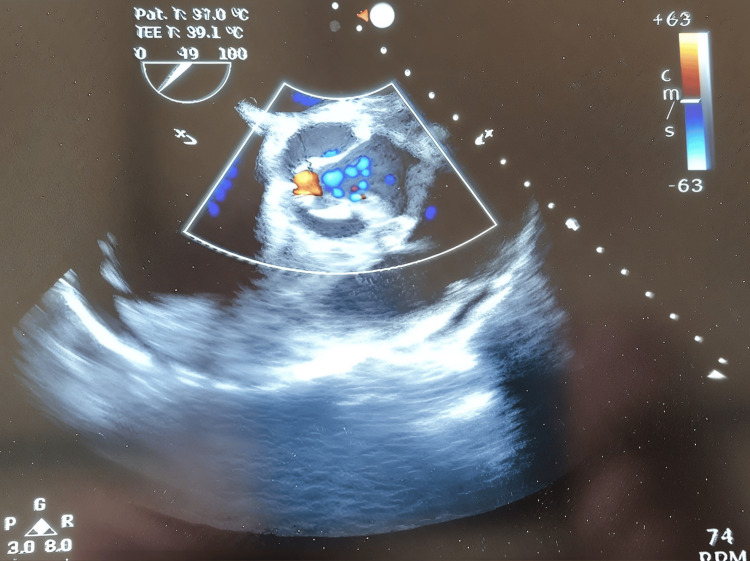
Aortic root abscess (as identified by perioperative transesophageal echocardiography).

**Figure 2:**
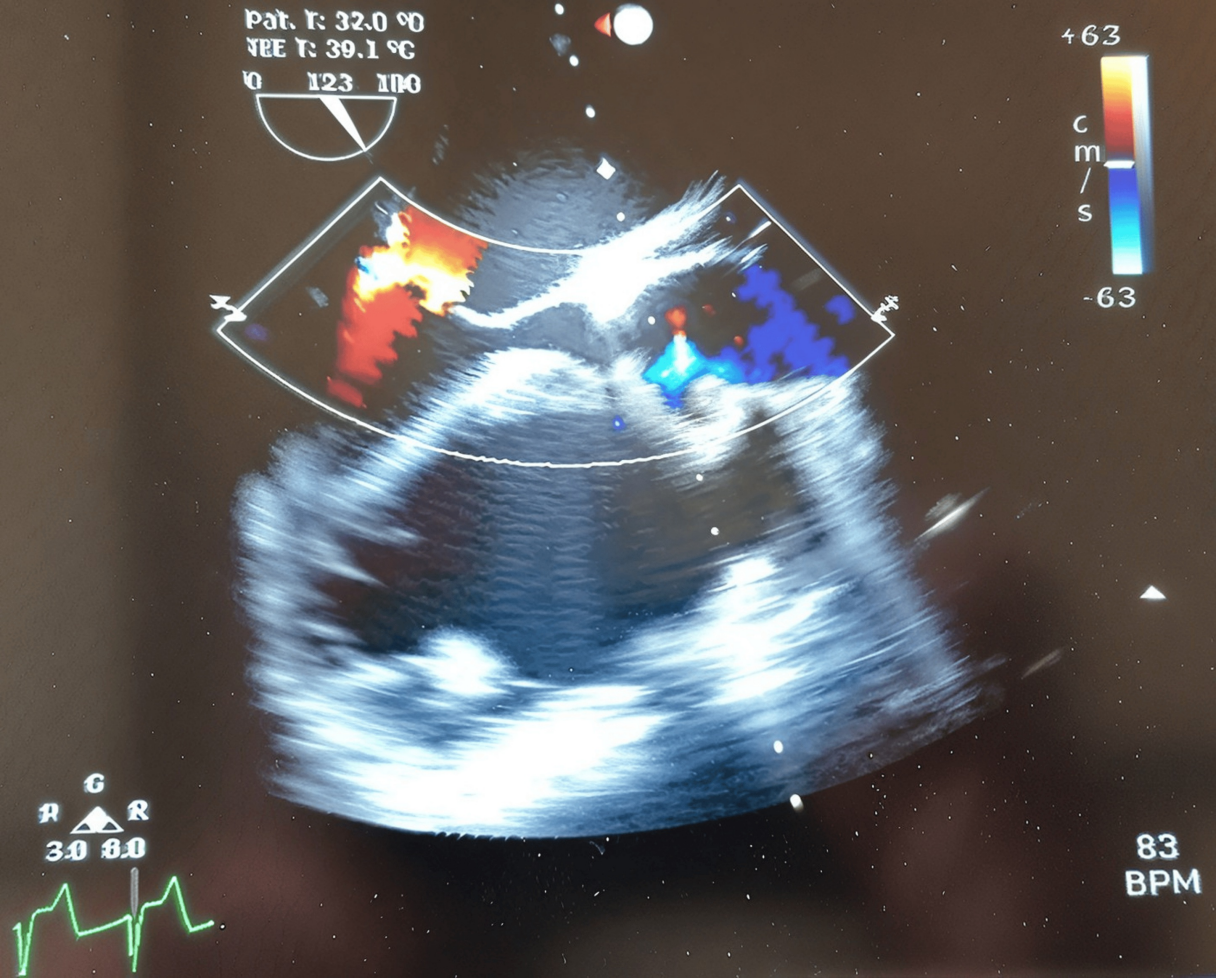
Aortic valve short axis with aortic regurgitation (as observed in our perioperative transesophageal echocardiography).

The patient’s medical history included intravenous drug use, HIV, hepatitis C infection, and type B lymphoma. Other comorbidities included severe chronic kidney failure, hypothyroidism, history of deep venous thrombosis, depression, chronic hyponatremia, and previous aortic valve and root replacement for endocarditis. His hematology history was significant for pancytopenia. His medication included allopurinol, bisoprolol, dapsone, emtricitabine/tenofovir alafenamide fumarate, dolutegravir, zopiclone, and levothyroxine. The patient presented with pyrexia, fatigue, and a septic profile. His vital signs were as follows: temperature 38.7 °C, blood pressure 90/50 mmHg, heart rate 105 beats per minute, and oxygen saturation 93%-94%. After diagnosing endocarditis and aortic root abscess with a heart ultrasound, he was admitted to the coronary care unit. Blood cultures confirmed bacteremia with Streptococcus bovis. Full blood count showed mild leukocytosis and thrombocytopenia (white blood cells, 15,000/μL; platelets, 46,000/μL; hemoglobin, 9.5 g/dL; C-reactive protein, 35 mg/L). There was a failure of early recognition of type B lymphoma during admission due to an inadequate and incomplete medical history, as well as the absence of any description in the electronic medical notes. After a thorough preanesthetic assessment, a hematology review was requested along with a platelet transfusion the evening before surgery. During transfusion, the patient became tachycardic and developed a generalized flushing. The administration was discontinued, and he was resuscitated with intravenous boluses of fluids, chlorphenamine, and hydrocortisone. No significant hemodynamic or airway compromise was observed. On the morning of surgery, a team briefing took place, and two hematology consultants - specializing in lymphoma and thrombosis - were available and recommended using washed platelets for transfusion. The anesthetic team performed testing of clotting and full blood count intraoperatively. It was recommended to transfuse two units of platelets before cardiopulmonary bypass (CPB). A joint decision was made for a test dose of 20 mL of FFP just before aortic cross-clamping to test for any allergic reaction. The platelet count fell from around 80,000 to below 15,000 before separating from CPB. In total, 6 g of tranexamic acid was given and an extra 100 mg of protamine on top of the reversal dose. After communication with the hematologist, four units of platelets, two pools of cryoprecipitate, and 15 mL/kg FFP were given. During the whole perioperative period, transesophageal echocardiography was performed to maximize the success of the operation (Figures 1-2). The procedure lasted eight hours, and the patient was transferred to the cardiac intensive care on a low dose of dobutamine and noradrenaline infusion. A central trunk rash was noted, and he received hydrocortisone 50 mg every six hours for the first 24 hours. He was extubated on Day 1 and, on Day 3, was transferred to the High Dependency Unit.

## Discussion

Platelet transfusion is common after aortic root replacement due to endocarditis. Platelet allergy is generally rare; however, platelets, following immunological or nonimmunological activation, may release biologically active mediators and actively participate in hypersensitivity reactions, as these cells express functional receptors for the Fc fragment of IgE. Alterations in platelet function have been observed in patients with both allergic and nonallergic hypersensitivity, including hypersensitivity to acetylsalicylic acid [2]. Patients with large B-cell lymphoma typically experience bone marrow suppression, as in our case, and a low platelet count has been previously highlighted [3].

Furthermore, platelet consumption occurred more rapidly than expected, and some authors suggest that activated platelets may contribute to transfusion reactions. Various hemostatic disturbances, particularly a drop in platelet count, were observed during anaphylactic shock [2]. FFP proved to be less reactive than platelets and caused minimal reaction, aside from the appearance of a postoperative rash. Postoperative tryptase levels were within the normal range. Fibrinogen levels are typically deranged and elevated in patients with diffuse large B-cell lymphoma, which is considered a poor prognostic factor [4]. Therefore, cryoprecipitate was not advised by the hematologist. Nonetheless, our patient had very low fibrinogen levels in the early post-CPB period. Aprotinin was avoided due to its higher incidence of allergic reactions, although a study involving 12,403 first exposures suggests that the risk is relatively low [5].

Thromboelastography (TEG) remains a useful tool for goal-directed transfusion, but it was not available on the day of the surgery. A meta-analysis by Wikkelsoe et al., including nine trials involving 776 participants, showed that there is weak evidence to support its use to guide transfusion in severe bleeding [6]. Eight trials involved cardiac surgery, and one trial investigated liver transplantations. No impact was identified on mortality, blood transfusions, incidence of reoperations, time to extubation, or length of stay in hospital and intensive care unit. The authors identified a significant reduction in blood loss favoring the use of TEG/rotational thromboelastometry (ROTEM), as well as a decrease in the proportion of patients receiving FFP and platelets (relative risk [RR] 0.39; 95% confidence interval [CI] 0.27-0.57) [6]. Finally, one could argue that the administration of even a minor bolus dose of FFP by the perfusionist on CPB before the aortic cross-clamp is uncommon, and no evidence in the literature was found to support this practice, except for the use of FFP instead of crystalloid for priming the CPB circuit in pediatric cardiac surgery [7].

## Conclusions

Redo surgery for infective endocarditis is characterized by extensive bleeding, and hemostasis remains a challenge, sometimes leading to high mortality. Allergic reaction to platelets is generally a rare entity. In our case, polytransfusion of blood products was required, particularly the administration of washed platelets throughout the entire perioperative period. The importance of a multidisciplinary approach was recognized early, as such a challenging case requires proactive management, effective communication, and collaboration between teams. Consequently, excellent coordination among clinicians was key to achieving a successful outcome.
